# Electrochemical Performance of Carbon-Rich Silicon Carbonitride Ceramic as Support for Sulfur Cathode in Lithium Sulfur Battery

**DOI:** 10.3390/nano12081283

**Published:** 2022-04-09

**Authors:** Fangmu Qu, Zhaoju Yu, Monika Krol, Nan Chai, Ralf Riedel, Magdalena Graczyk-Zajac

**Affiliations:** 1 Institut für Materialwissenschaft, Technische Universität Darmstadt, Otto-Berndt-Straße 3, 64287 Darmstadt, Germany; m.graczyk-zajac@enbw.com (M.G.-Z.); monika.krol@aalto.fi (M.K.); n.chai@materials.tu-darmstadt.de (N.C.); riedel@materials.tu-darmstadt.de (R.R.); 2 Key Laboratory of High-Performance Ceramic Fibers, Ministry of Education, College of Materials, Xiamen University, Xiamen 361005, China; 3 Fujian Key Laboratory of Advanced Materials, College of Materials, Xiamen University, Xiamen 361005, China; 4 EnBW Energie Baden-Württemberg AG, Durlacher Allee 93, 76131 Karlsruhe, Germany

**Keywords:** SiCN ceramic matrix, disordered carbon, porous structure, sulfur cathode

## Abstract

As a promising matrix material for anchoring sulfur in the cathode for lithium-sulfur (Li-S) batteries, porous conducting supports have gained much attention. In this work, sulfur-containing C-rich SiCN composites are processed from silicon carbonitride (SiCN) ceramics, synthesized at temperatures from 800 to 1100 °C. To embed sulfur in the porous SiCN matrix, an easy and scalable procedure, denoted as melting-diffusion method, is applied. Accordingly, sulfur is infiltrated under solvothermal conditions at 155 °C into pores of carbon-rich silicon carbonitride (C-rich SiCN). The impact of the initial porosity and microstructure of the SiCN ceramics on the electrochemical performance of the synthesized SiCN-sulfur (SiCN-S) composites is analysed and discussed. A combination of the mesoporous character of SiCN and presence of a disordered free carbon phase makes the electrochemical performance of the SiCN matrix obtained at 900 °C superior to that of SiCN synthesized at lower and higher temperatures. A capacity value of more than 195 mAh/g over 50 cycles at a high sulfur content of 66 wt.% is achieved.

## 1. Introduction

The rapidly growing technological development of the modern world relies on the increasing demand for the allocation of energy, in the form of electrical power. While renewable energies are continuously growing worldwide, fossil fuels still show an increasing consumption associated with the production of high levels of carbon dioxide and other greenhouse gasses, which are responsible for our climate change [1]. In order to significantly reduce the usage of fossil fuels and, at the same time, increase the production of electrical power, renewable energies have to be further developed in the near future. One of the most sustainable solutions is using renewable green sources based on wind, water, or solar energy, which are clean and do not emit as much greenhouse gasses. However, renewable sources are intermittent and require efficient energy storage systems to become a valid competitor of fossil fuels. Electrochemical energy storage systems such as the Li-ion battery (LIB) represent the most promising technology to fill the gap between energy production and utilization securing energy supply. However, there are increasing concerns regarding the sustainability and criticality of materials used in LIBs, such as cobalt-containing cathode materials [2]. One of the most promising alternative Li-ion-based battery systems is the lithium-sulfur technology. Sulfur is abundant and cheap—it is considered waste by industries. Additionally, Li-S with pristine sulfur cathode yields superior high theoretical capacity (1672 mAh/g) and gravimetric energy (2500 Wh/kg) [3]. Nevertheless, the electrically insulating nature of sulfur and lithium sulfide, its poor cycling performance, due to the high polysulfides solubility and large volume (80%) changes during the redox reaction, impede the use of sulfur as cathode material. Thus, designing a highly efficient Li-sulfur system still remains a challenge.

For tackling above issues, various strategies have been suggested. Providing skeleton materials with porous structure to reduce the “shuttle effect” and volume expansion of sulfur, as well as incorporating conductive materials to increase the electrical conductivity, are the main methods for enhancing the electrochemistry performance of lithium-sulfur batteries (LSB) [4,5,6,7]. In the past decade, nanomaterials, such as graphene, carbon nanotubes, porous carbon, and SiC, as well as nitrogen-doped carbonaceous materials, are widely investigated as skeleton and conductive materials for Li-S batteries, and they indeed lead to an improvement of the electrochemical performance of the sulfur cathode [8,9,10,11,12,13,14,15,16,17]. However, most of the applied nanomaterials are expensive in production; therefore, their practical large-scale commercialization is hard to achieve. For normal porous carbon materials, their soft structure is not enough to hamper the volume expansion during the battery cycling process. Thus, there is still a big demand for the development of an advanced host material, which is not only mechanically flexible and robust, but also shows enhanced conductivity and can be produced at low costs.

C-rich polymer derived ceramics (PDCs) are remarkable materials, due to their robust architecture, suitable to restrain volume changes and tunable electrical conductivity providing pathways for electrons and ions [18,19,20,21,22]. In addition, their porous property makes it possible to load sulfur and to realize physical adsorption barrier for polysulfides to hamper the “shuttle effect” [23,24,25,26]. Our previous work showed that porous C-rich SiCN ceramics pyrolyzed at temperatures between 1000 °C and 1600 °C can stabilize the electrochemical behaviour of sulfur [27]. It has been also demonstrated that the morphology of C-rich SiCN ceramics significantly affects the electrochemical performance of the S/SiCN composites and nitrogen contained in the ceramic matrix has a stabilizing effect for the electrochemical performance. Our former study [28,29] also showed that the microstructure of SiCN ceramics processed at temperatures lower than 1000 °C significantly differs from those prepared at higher temperatures. Thus, in this work, we further investigate the electrochemical stability of SiCN-S composites containing C-rich SiCN ceramics pyrolyzed at temperatures below 1000 °C in more detail. The C-rich SiCN-S composites are synthesized using a scalable melting-diffusion method [27]. After the melting-diffusion procedure, sulfur is introduced and embedded into the C-rich SiCN ceramic. The obtained SiCN-S composites are characterized by XRD, scanning electron microscopy, Raman spectroscopy, and N_2_ adsorption, followed by their electrochemical performance evaluation.

## 2. Experimental

### 2.1. Synthesis of C-Rich SiCN Ceramics

**Scheme 1:**
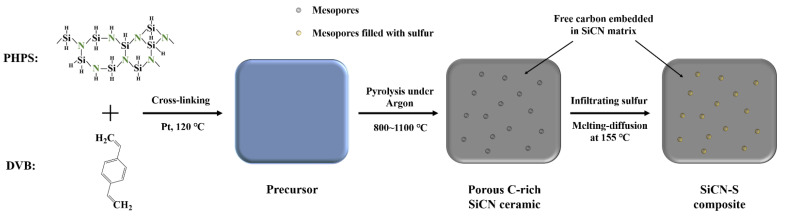
Preparation of the C-rich SiCN-S composite.

Cross-linking reactions of commercial perhydropolysilazane (PHPS, DURAZANE 2250, Merck Performance Materials GmbH, Wiesbaden, Germany) with divinylbenzene (DVB, 80%, mixture of isomers, Sigma-Aldrich, Burlington, MA, USA) in Di-n-butylether (DBE, Merck Performance Materials GmbH, Wiesbaden, Germany) as the solvent and catalyzed by platinum (0)-1,3-divinyl-1,1,3,3-tetramethyldisiloxane complex solution (~Pt 2% in xylene, Sigma-Aldrich, Burlington, MA, USA) were performed via the same method, as described in our previous work—reference [27]. The obtained colorless solid precursors were then pyrolyzed in a Schlenk tube under argon flow at 800 °C, 900 °C, and 1100 °C. Finally, the C-rich SiCN ceramic matrices were denoted as SiCN-800, SiCN-900, and SiCN-1100. The schematic diagram of the preparation of the C-rich SiCN-S composite is shown in Scheme 1.

### 2.2. Fabrication of SiCN-S Composites

Firstly, sulfur (Sigma-Aldrich, Burlington, MA, USA) and the obtained C-rich SiCN ceramic matrices were mixed via ball milling for 20 min, with a mass ratio of 2:1 (66.6 wt% of sulfur). Secondly, the obtained mixtures were heated to 155 °C in an autoclave, with Teflon inlet, for 24 h. Finally, the obtained samples (SiCN-S-800, SiCN-S-900, and SiCN-S-1100) were powderized by grounding them in a mortar.

### 2.3. Characterization of the Samples

Crystalline phases of the samples were measured by high power x-ray diffraction (STOE STADIP, Darmstadt, Germany) with monochromatic Mo-K_α_ radiation at a 2θ range of 5° to 45°. Specific surface area and pore size distribution of the C-rich SiCN ceramics were determined by N_2_ adsorption and desorption measurements at 77 K (Quantachrome Autosorb-3B, Boynton Beach, FL, USA). Raman spectra were detected via a micro-Raman spectrometer (Horiba HR800, Kyoto, Japan) with an Ar-ion laser with the wavelength 514.5 nm between a scan window of 0 cm^−1^ to 4000 cm^−1^. All filters and parameters were kept constant for measurements. Morphology images and elemental mapping analysis of the samples were performed by scanning electron microscopy on a Philips XL30 FEG (Koninklijke Philips N.V., Amsterdam, Netherlands).

### 2.4. Electrochemical Measurement

Galvanostatic Cycling was performed by using a VMP-multipotentiostat (Biologic Science Instruments, Seyssinet-Pariset, France), at a controlled constant temperature of 25 °C. All cells were tested in a voltage window between 1.2 and 3.6 V under a constant current density of 83.5 mA/g. Cyclic Voltammogram was recorded at a scan rate of 0.02 mV/s. For electrodes fabrication, SiCN-S composites, polyvinylidenflouride (PVDF, Sigma-Aldrich, Burlington, MA, USA), and carbon black (Super P, Timcal Ltd., Bodio, Switzerland), with a mass ratio of 85 wt%, 10 wt%, and 5 wt%, were mixed in N-Methyl-2-pyrrolidone (NMP, BASF, Ludwigshafen, Germany) to form a slurry. A doctor blade was used to paint an aluminum foil with the obtained slurry. The coated aluminum foil was dried at 40 °C for 24 h to evaporate the solvent. After cutting, the samples were further dried for 24 h in a Buchi furnace (Labortechnik AG, Essen, Germany). Finally, nummular electrode slices with 10 mm in diameter were obtained. All electrodes were assembled in Swagelok cells, under argon, in a glovebox. Lithium foil (0.75 mm thickness, Alfa Aesar GmbH & Co KG, Karlsruher, Germany) was used as counter electrode and QMA (Whatman, Maidstone, UK) was used as separator; 1,3-dioxolane (DOL, Sigma-Aldrich, Burlington, MA, USA) and dimethoxymethane (DME, Sigma-Aldrich, Burlington, MA, USA) (volume ratio: 1:1), with 1 M lithium bis(trifluoromethanesulfonyl)imide (LiTFSI, Sigma-Aldrich, Burlington, MA, USA), were used as electrolyte (containing 0.1 M LiNO_3_ for alleviating the “shuttle effect”).

## 3. Results and Discussion

### 3.1. X-ray Diffraction

**Figure 1:**
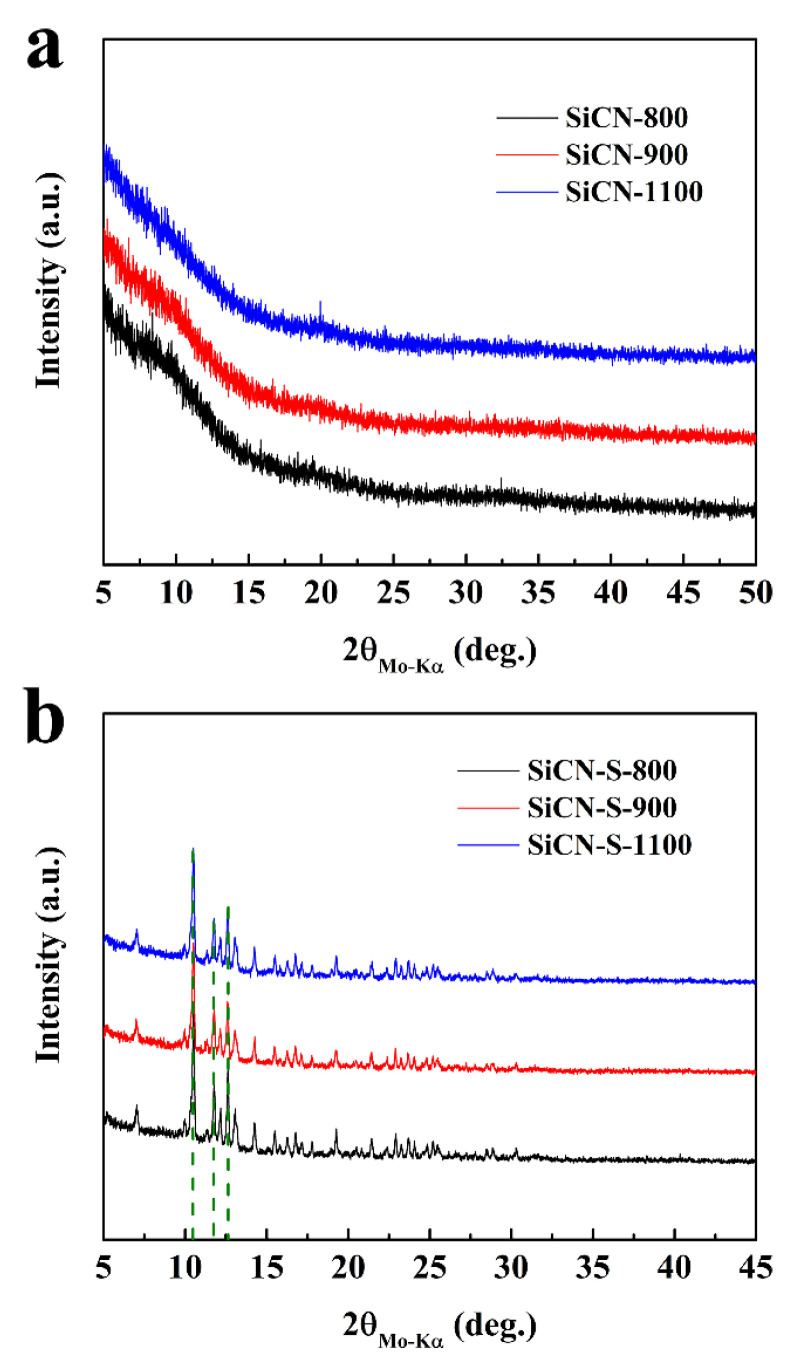
XRD patterns of (**a**) C-rich SiCN ceramics and (**b**) SiCN-S composites processed at different pyrolysis temperatures. The green dotted line in Figure 1b represents the position of the characteristic peaks of S_8_.

The XRD patterns of C-rich SiCN ceramics and SiCN-S composites are shown in Figure 1. The diffractograms shown in Figure 1a reveal a characteristic amorphous structure of the synthesized SiCN ceramics at all temperatures, indicating that no crystallization occurs in the pyrolysis temperature range between 800 °C and 1100 °C. Figure 1b presents the diffraction patterns, after introducing sulfur into the SiCN ceramic matrix via solvothermal treatment in an autoclave. All the diffraction lines are associated with crystallized sulfur (S_8_, reference card number: [01-085-0799]). 

### 3.2. N_2_ Adsorption-Desorption Measurements

**Figure 2:**
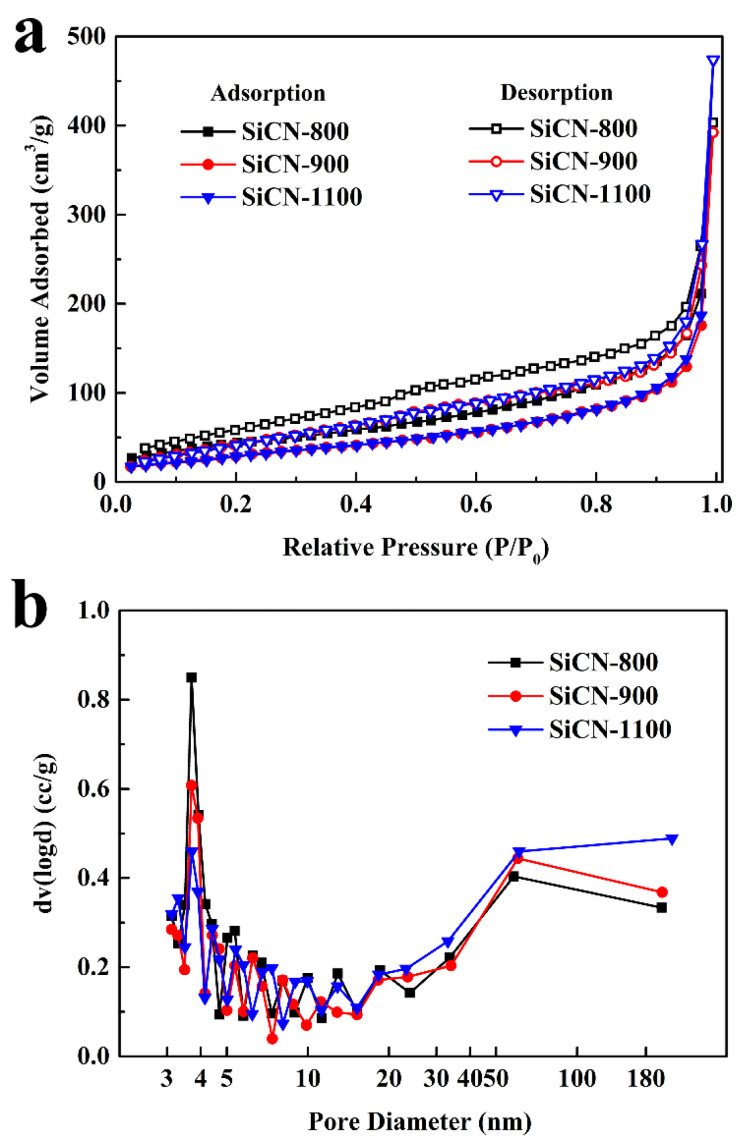
N_2_ adsorption-desorption isotherms (**a**) and pore diameter distribution (**b**) of C-rich SiCN ceramic matrix.

Nitrogen adsorption-desorption measurement was performed to determine the specific surface area (SSA) and to provide insights into the pore size and distribution. The N_2_ adsorption-desorption isotherms are depicted in Figure 2a. A hysteresis loop is observed at P/P_0_ ≈ 0.5, due to capillary condensation of nitrogen in mesopores, especially for the sample SiCN-S-800 [30]. It indicates that all samples exhibit mesoporous character. Detailed SSA data and pore structure parameters, such as total pore volume (Vt) and isotherm type and average pore diameter (APD), are listed in Table 1. All the isotherms are described by type IV behavior, which is characteristic for materials containing mesopores [31]. From 800 °C to 900 °C, SSA and V_t_ of SiCN ceramics decrease with increasing pore size (corresponding to the value of APD). Due to equipment precision, larger mesopores could not be detected by a BET approach. With further increasing of the pyrolysis temperature to 1100 °C in argon, V_t_ and APD turn to increase, but there is no obvious change in SSA. The change in APD indicates a reduced the presence of bigger pores in the sample pyrolyzed at 1100 °C. The pore size distribution of all ceramic samples is presented in Figure 2b. With increasing pyrolysis temperature, the amount of mesopores in the range between 3 and 4 nm decreases.

### 3.3. Raman Spectroscopy

**Figure 3:**
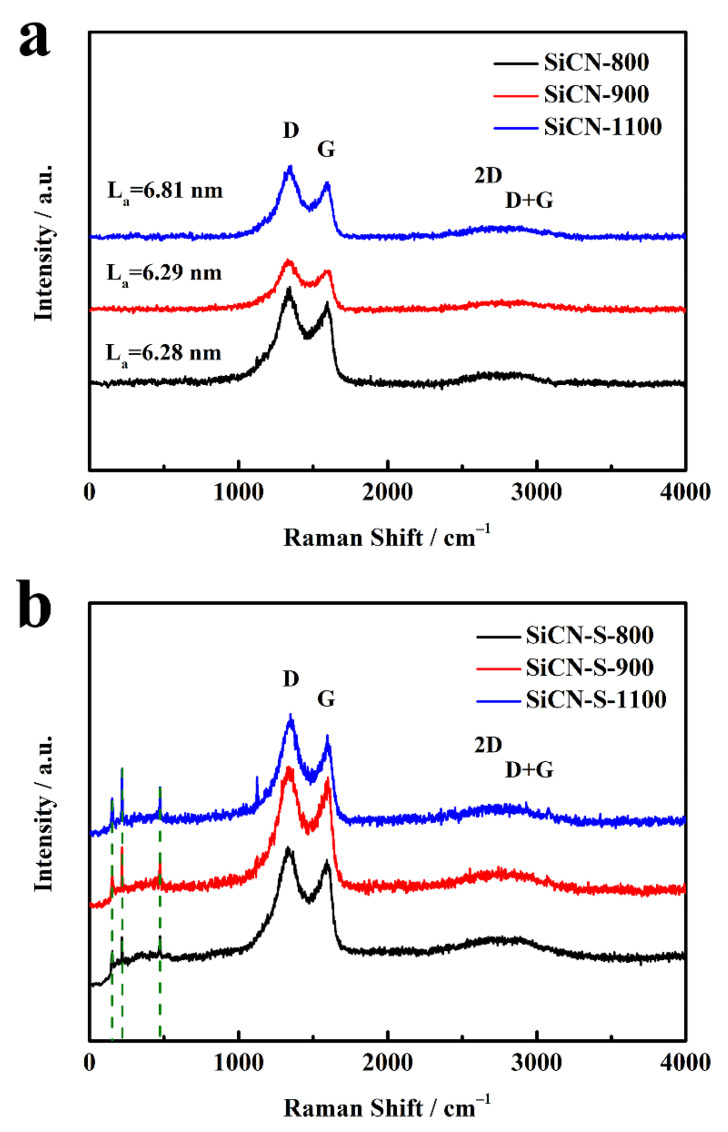
Raman spectra of C-rich SiCN ceramic matrices (**a**) and SiCN-S composites (**b**). The green dotted line in Figure 3b represents the position of the characteristic bands of S_8_.

Figure 3 presents the Raman spectra of our SiCN ceramics without and with sulfur. All samples show characteristic D band and G band peaks of carbon at around 1345 cm^−1^ and 1583 cm^−1^, as well as a broad peak between 2500 cm^−1^ and 3000 cm^−1^, corresponding to the 2D and D + G band of carbon, respectively. The sulfur-free SiCN ceramics show decreased ratio of I (A_D_)/I (A_G_) and increased crystallite size (L_a_), with increasing pyrolysis temperature (Figure 3a and Table 1) [27]. The I (A_D_)/I (A_G_) ratio of all samples indicate that the carbon inside the SiCN ceramics exhibits amorphous structure originating from disordered sp^2^-hybridized carbon. After introducing sulfur into the SiCN ceramics, characteristic bands of sulfur are also detected in the Raman spectra besides that of the D, G, 2D, and D + G bands of carbon (Figure 3b).

### 3.4. Scanning Electron Microscopy Measurements

**Figure 4:**
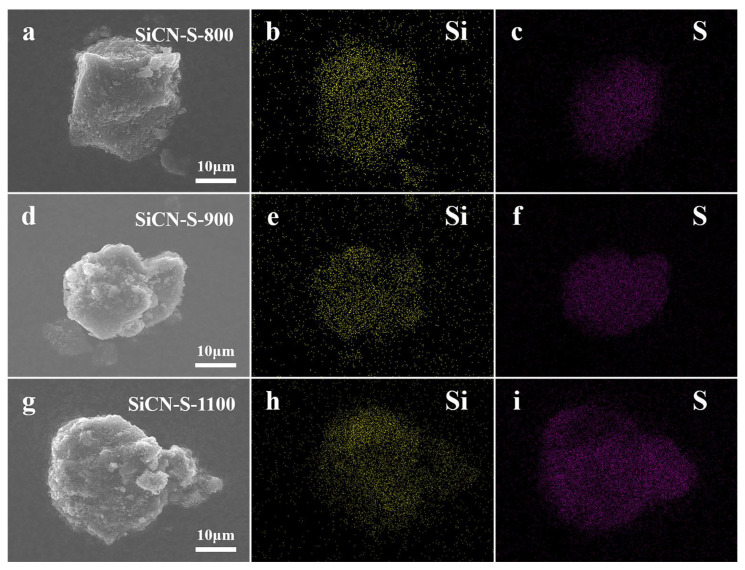
SEM images and EDS elemental mapping of SiCN-S-800 (**a**–**c**), SiCN-S-900 (**d**–**f**), SiCN-S-1100 (**g**–**i**). Yellow represents Si, purple represents S.

Figure 4 presents the morphologies and elemental mapping results of all SiCN-S composites, as measured by scanning electron microscopy (SEM) and energy dispersive spectroscopy (EDS). The synthesized SiCN-S composite particles exhibit a size between 20–30 μm and a rough surface morphology. The EDS results show that sulfur is uniformly distributed in the SiCN material. This result clearly shows that the autoclave technique applied to load sulfur in SiCN is highly efficient and allows for uniform embedding of sulfur into the porous structure of the SiCN matrix. 

### 3.5. Galvanostatic Charge/Discharge Measurements

**Figure 5:**
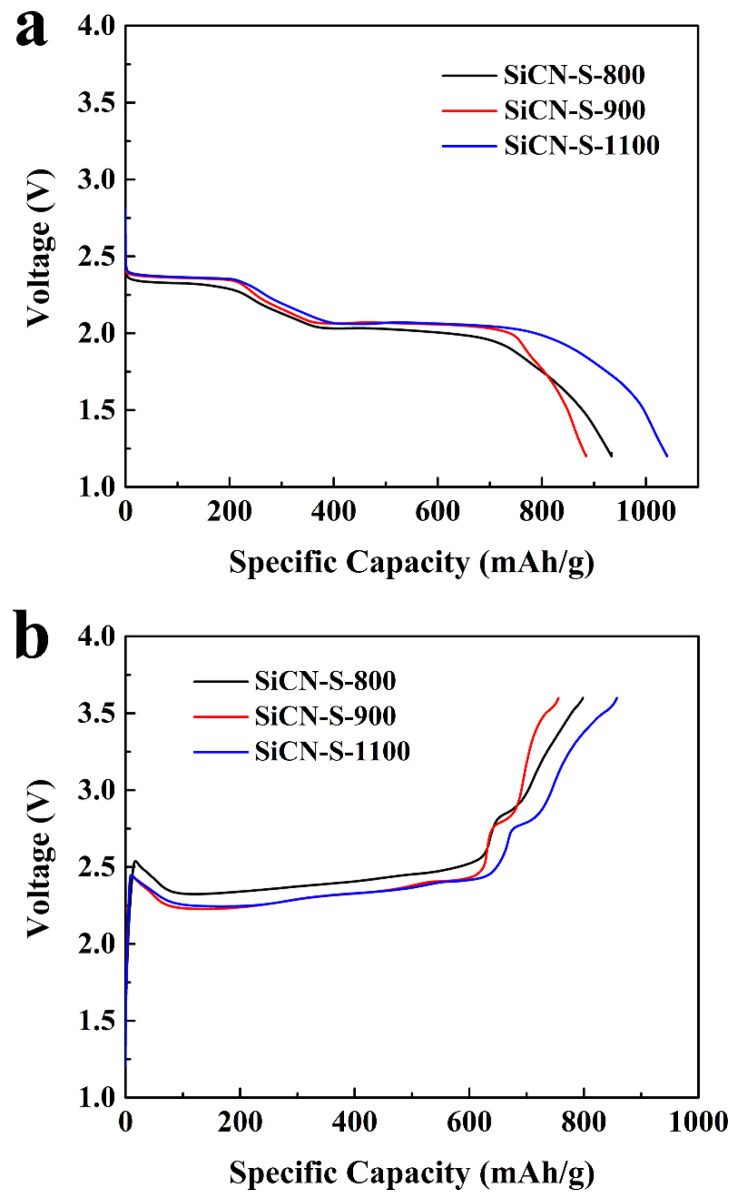
Lithiation curve of samples SiCN-S-800, SiCN-S-900, and SiCN-S-1100 (**a**); delithiation curve of samples SiCN-S-800, SiCN-S-900, and SiCN-S-1100 (**b**).

Initial lithiation/delithiation curves of the SiCN-S composites are shown in Figure 5. Two typical lithiation voltage plateaus of sulfur are clearly visible at around 2.4 and 2.0 V during the lithiation process. This behavior is related to the reaction of S_8_ with Li to form soluble polysulfides Li_2_S_x_ (4 ≤ x ≤ 8) and solid Li_2_S_2_ and Li_2_S [6,15,33]. The relevant reaction equations are as follows [34]:

(1) $$S_{8}+2e^{-}+2{\mathrm{Li}}^{+}⟶{\mathrm{Li}}_{2}S_{8}$$

(2) $${\mathrm{Li}}_{2}S_{8}+2e^{-}+2{\mathrm{Li}}^{+}⟶2{\mathrm{Li}}_{2}S_{4}$$

(3) $$2{\mathrm{Li}}_{2}S_{4}+4e^{-}+4{\mathrm{Li}}^{+}⟶4{\mathrm{Li}}_{2}S_{2}$$

(4) $$4{\mathrm{Li}}_{2}S_{2}+8e^{-}+8{\mathrm{Li}}^{+}⟶8{\mathrm{Li}}_{2}S$$

Nevertheless, the two voltage plateaus of SiCN-S-800 (2.33 and 2.03 V) reveal a hysteresis, compared with other two composites (2.36 and 2.06 V), relating to a higher polarization [35,36]. This phenomenon is also seen in the delithiation curves (2.35 V of SiCN-S-800 vs. 2.23 V of SiCN-S-900 and SiCN-S-1100), which is discussed in terms of sample SiCN-S-800, which has the lowest degree of graphitization among all composites based on the Raman results. This, in turn, results in the lowest conductivity of the free carbon phase in sample SiCN-S-800 [37]. Initial lithiation capacities and corresponding columbic efficiencies of all SiCN-S composites are listed in Table 2. The initial lithiation capacity decreases from sample SiCN-S-800 to sample SiCN-S-900 and then increases in sample SiCN-S-1100. This behavior is explained by the amount of accessible sulfur embedded in the ceramic matrix, which decreases from SiCN-S-800 to SiCN-S-900 with decreasing SSA of the ceramic matrix. With increasing pyrolysis temperature, the degree of graphitization increases, resulting in an enhanced electrical conductivity of the SiCN matrix and, hence, higher initial lithiation capacity of the sulfur. All composites exhibit around 85% of initial coulombic efficiency, which indicates that the characteristic “shuttle effect” is less pronounced in all samples in the beginning of cycling.

### 3.6. Cyclic Voltammperometry

**Figure 6:**
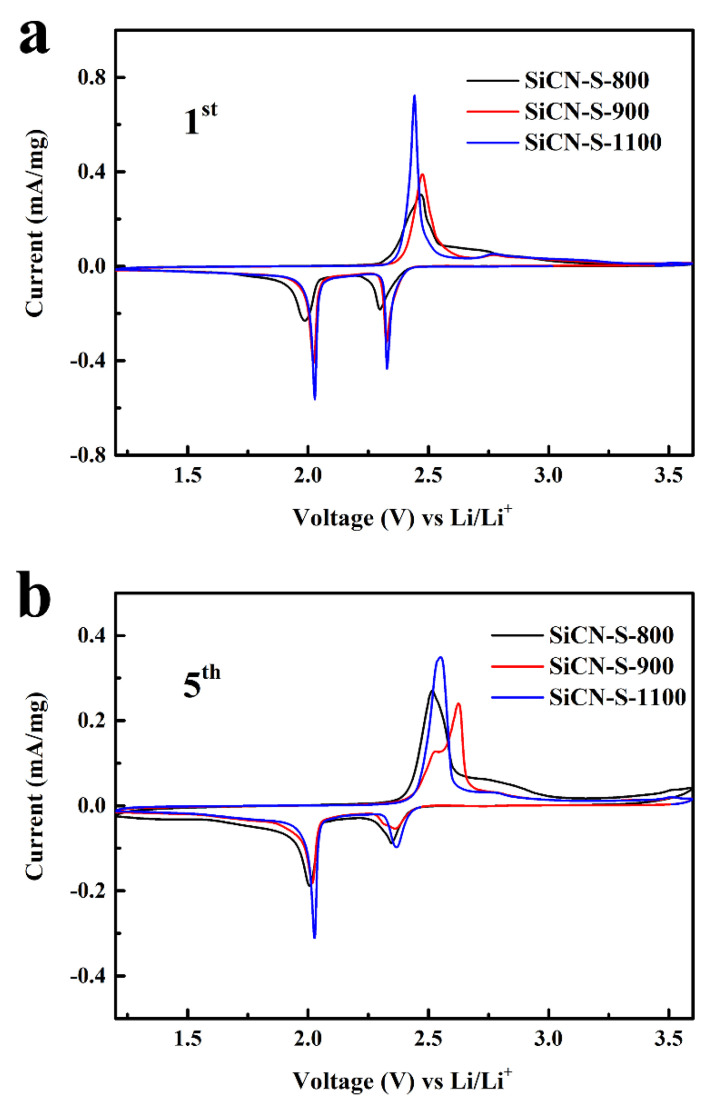
Cyclic voltammograms of samples SiCN-S-800, SiCN-S-900, and SiCN-S-1100 at a scan rate of 0.02 mV/s. The first cycle (**a**) and the fifth cycle (**b**).

To follow the redox reactions in the investigated system in more detail, cyclic voltammperometry measurements have been performed and are shown in Figure 6. For the initial cycle of all composites, the curves present two cathodic peaks related to the lithiation of S_8_ to higher order soluble polysulfides (Li_2_S_x_ (4 ≤ x ≤ 8)) and, finally, to solid Li_2_S_2_ and Li_2_S, as well as one anodic peak due to the conversion of polysulfides back to elemental sulfur S_8_. It is worth noting that the cathodic peak appears at higher potential position also with higher intensity for the SiCN-S composites pyrolyzed at 1100 °C. This feature, again, indicates that the electrical conductivity of the C-rich SiCN matrix increases, leading to a higher sulfur capacity. Besides, there is a significant increase of the distance between the cathodic and the anodic peak for the sample SiCN-S-800 during the first CV cycle. The cathodic peaks are found at 2.30 and 1.99 V, whereas for the SiCN-S-900 and SiCN-S-1100 composites they are located at 2.33 and 2.03 V, respectively. The anodic peaks of the samples SiCN-S-800 and SiCN-S-900 are at around 2.47 V, whereas for SiCN-S-1100 the anodic peak is shifted to 2.44 V. This feature is caused by the higher ohmic resistance in the case of the samples synthesized at lower temperature [35,38,39]. Additionally, it is not more visible, in case of SiCN-S-900, meaning that, in this case, a degree of graphitization of the free carbon phase is enough to provide a sufficient electrical conductivity. After the fifth cycle, the ohmic resistance of SiCN-S-800 and SiCN-S-900 is still a bit higher than that of SiCN-S-1100, but much lower than for the initial cycle. Both cathodic and anodic peak intensity of the samples SiCN-S-1100 decrease compared with the samples SiCN-S-800 and SiCN-S-900. This behavior reveals that the cyclic stability of the electrode material is significantly decreased when the pyrolysis temperature is beyond 900 °C.

### 3.7. Extended Cycling Performance 

**Figure 7:**
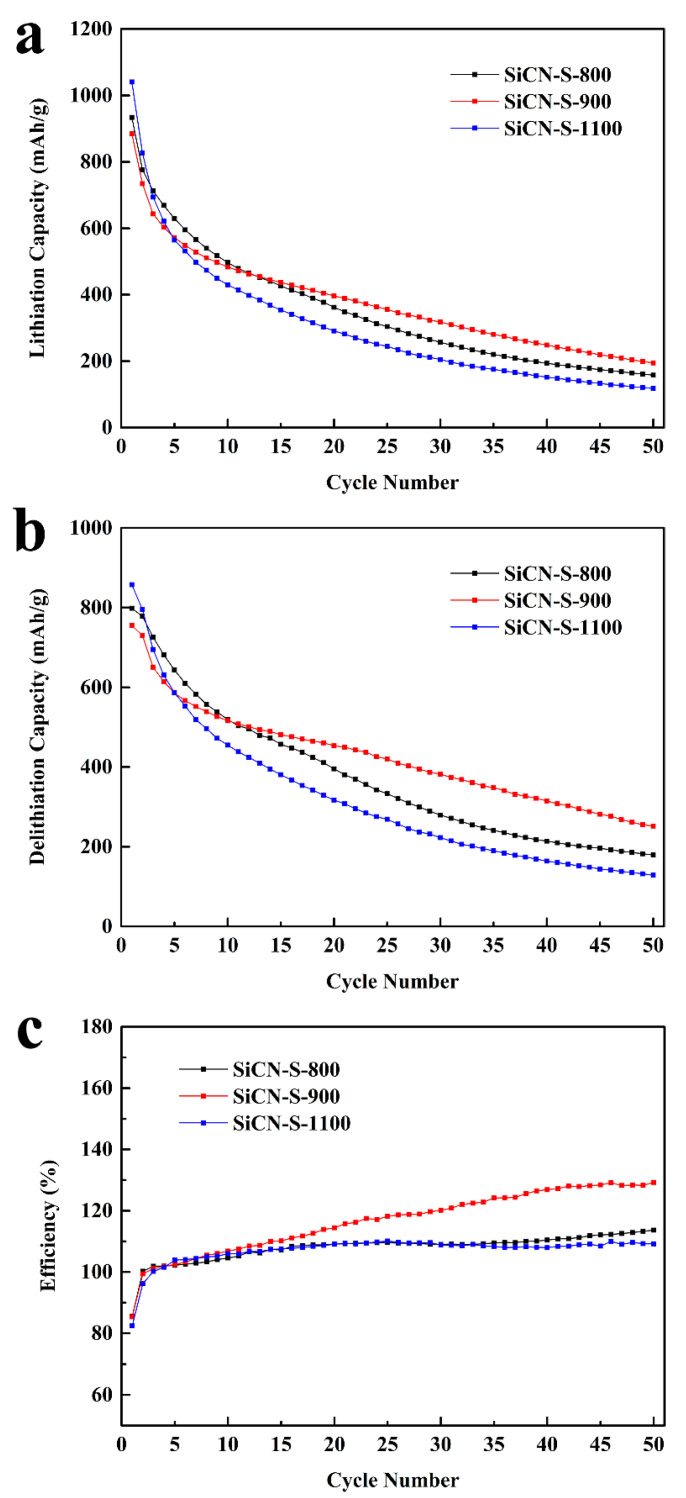
Cycling performance of samples SiCN-S-800, SiCN-S-900, and SiCN-S-1100. Lithiation capacity (**a**), delithiation capacity (**b**), and columbic efficiency (**c**).

Figure 7a–c represents the lithiation/delithiation specific capacity and corresponding coulombic efficiency of all SiCN-S composites. Besides the initial lithiation capacity, the reversible capacity after 50 cycles, the coulombic efficiency and capacity retention of all SiCN-S composites are listed in Table 2. As shown in Figure 7a,b, both the lithiation and delithiation capacity of the sample SiCN-S-900 are the lowest in the first four cycles. Nevertheless, after the fourth cycle, its specific capacity surpasses that of the sample SiCN-S-1100. After 12 cycles, the sample SiCN-S-900 even surpasses that of the SiCN-S-800 and remains the highest capacity until the 50th cycle. The lowest performance is registered for the sample SiCN-S-1100. In the end, after 50 cycles, the sample SiCN-S-900 exhibits the highest lithiation retention of 22%. The enhancement of the electrochemical performance of sample SiCN-S-900, in comparison to that of SiCN-S-800, is mainly due to the enhanced conductivity resulted from the increasing of the degree of graphitization of the free carbon. Although the SSA of the sample SiCN-900 is lower, its total pore volume V_t_ changed little, in comparison with the sample SiCN-800, and it reveals more mesopores, in comparison to SiCN-1100 (results of BET). The presence of mesopores is of advantage for stabilization of sulfur electrochemical behavior [27,40,41], which can explain the best electrochemical performance of SiCN-S-900 among measured samples. Even though SiCN-S-1100 contains more organized free carbon phase and thus reveals higher electronic conductivity, SiCN-S-900 outperforms it in prolonged cycles stability. It is known that the “shuttle effect” originating from the diffusion of soluble polysulfides usually leads to a coulombic efficiency higher than 100% [11,33,42]. This phenomenon was also discussed in our previous work. During delithiation process, the long-chain soluble polysulfides pass through the membrane and migrate to Li anode, redox reaction happens, and short chain polysulfides are formed; then, the short-chain polysulfides migrate back and are re-oxidized to long-chain polysulfides, resulting in long delithiation process and excessive coulombic efficiency over 100% [27]. Hence, the level of coulombic efficiency can reflect the degree of the “shuttle effect”. While, in addition to the remarkable initial coulombic efficiency of all SiCN-S composites, in the first 14 cycles, all samples keep the state where the coulombic efficiency is not growing rapidly (lower than 110%). In the subsequent cycles up to the 50th cycle, all samples except SiCN-S-900 exhibit a coulombic efficiency around 110%. This finding in turn demonstrates that that the lowest extent of a “shuttle effect” is observed for SiCN-S-800 and SiCN-S-1100. As for the sample SiCN-S-900, its coulombic efficiency, growing from 110% to 130%, is a sign of a progressing polysulfides diffusion (“shuttle effect”), visible also in an excessive anodic polarization of the cyclic voltammetry curve shown in Figure 6b.

In our former study, the sample SiCN-S-1000 (pyrolyzed at 1000 °C) was identified as the sample with the most promising electrochemical behavior [27]. The screening of this low pyrolysis temperature region, performed in this work, allowed us to find out the tendency in electrochemical behavior. SiCN-S-900 composite electrode demonstrates a similar electrochemical stability to SiCN-S-1000. Both materials contain amorphous, defective free carbon, organized enough to provide a sufficient electrical conductivity, and dispersed in the SiCN matrix, with significant amount of mesoporous present in their microstructure, thus providing a stabilizing host for sulfur infiltration. At higher pyrolysis temperature, more ordered free carbon phase is present, however a pore diameter increase, resulting in lower capacity retention. At lower pyrolysis temperatures, the conductivity is provided by a free carbon phase exclusively, this phase however is not able to provide the robustness, leading to enhanced shuttling effect after 20 cycles. The presence of nitrogen in the electrode materials produced at lower pyrolysis temperature appears to be of advantage for polysulfides stabilization [43]. Therefore, a trade-off between the choice of the pyrolysis temperature and the effects of it on conductivity, capacity, and cycling stability should always be considered carefully. In addition, the mesoporous morphology of the SiCN sulfur support is beneficial for the cathode stabilization, but it is not a necessary condition, since other properties, such as SSA, degree of graphitization, etc., play a crucial role.

## 4. Conclusions

A series of C-rich SiCN ceramics, pyrolyzed at temperatures ≤ 1100 °C, were synthesized to investigate the effect of the corresponding change of microstructure on the electrochemical performance, when used as cathode material in a Li-S battery. The melting-diffusion method has been applied for sulfur infiltration into the SiCN ceramic matrices. The resulting SiCN-S composites have been characterized as a sulfur host for Li-S batteries. Their electrochemical performance has been analyzed, with respect to the initial microstructure and composition of the SiCN ceramic. The results indicate that the composite electrode, comprised of the SiCN matrix synthesized at 900 °C (sample SiCN-S-900), possesses enhanced electrochemical stability and higher capacity (more than 195 mAh/g over 50 cycles) at a high sulfur content of 66 wt.% than that of the cathode materials containing SiCN ceramics produced at lower and higher temperatures. The superior electrochemical performance of sample SiCN-S-900 is attributed to the microstructural integrity of the electrode produced at lower temperature, in line with higher composite conductivity.

## Figures and Tables

**Table 1:** Pore structure parameters analyzed by BET and characterization of the developed free carbon measured by Raman spectroscopy. Specific surface area (SSA), total pore volume (V_t_), average pore diameter (APD), intensity ratio of I (A_D_)/ I (A_G_), and crystallite size (L_a_) of samples SiCN-800, SiCN-900, and SiCN-1100 (Gaussian-type curve fitting applied [32]).

Sample	SiCN-800	SiCN-900	SiCN-1100
SSA (m^2^/g)	162	116	115
V_t_ (cm^3^/g)	0.62	0.61	0.73
Isotherm Type	IV	IV	IV
APD (nm)	15.46	20.94	25.5
I (A_D_)/I (A_G_) ratio	2.68	2.67	2.47
L_a_ (nm)	6.28	6.29	6.81

**Table 2:** Electrochemical parameters of the sulfurized SiCN samples.

Samples	Initial Lithiation Capacity (mAh/g)	Reversible Capacity after 50 Cycle (mAh/g)	Initial Coulombic Efficiency (%)	Capacity Retention (%)
SiCN-S-800	934	158	85	17
SiCN-S-900	885	195	85	22
SiCN-S-1100	1040	118	82	11

## Data Availability

Not applicable.
